# Impact of Carbon Source on Bacterial Cellulose Network Architecture and Prolonged Lidocaine Release

**DOI:** 10.3390/polym16213021

**Published:** 2024-10-28

**Authors:** Julia Amorim, Kuotian Liao, Aban Mandal, Andréa Fernanda de Santana Costa, Eleftheria Roumeli, Leonie Asfora Sarubbo

**Affiliations:** 1 Rede Nordeste de Biotecnologia (RENORBIO), Universidade Federal Rural de Pernambuco (UFRPE), Rua Dom Manuel de Medeiros, s/n—Dois Irmãos, Recife 52171-900, PE, Brazil; julia.didier@ufrpe.br; 2 Department of Materials and Science and Engineering, University of Washington (UW), 2110 Mason Road, Roberts Hall 302, Seattle, WA 98195, USA; timliao@uw.edu (K.L.); abanm@uw.edu (A.M.); 3 Instituto Avançado de Tecnologia e Inovação (IATI), Rua Potyra, n. 31, Prado, Recife 50751-310, PE, Brazil; andrea.santana@ufpe.br; 4 Centro de Design Comunicação, Campus Acadêmico da Região Agreste, Universidade Federal de Pernambuco (UFPE), Av Marielle Franco, s/n—Nova Caruaru, Caruaru 50670-900, PE, Brazil; 5 Escola de Tecnologia e Comunicação, Universidade Católica de Pernambuco (UNICAP), Rua do Príncipe, n. 526, Boa Vista, Recife 50050-900, PE, Brazil

**Keywords:** biomaterials, bacterial cellulose, nanocellulose, drug delivery, hydrogel, green nanomaterials

## Abstract

The biosynthesis of bacterial cellulose (BC) is significantly influenced by the type of carbon source available in the growth medium, which in turn dictates the material’s final properties. This study systematically investigates the effects of five carbon sources—raffinose (C_18_H_32_O_16_), sucrose (C_12_H_22_O_11_), glucose (C_6_H_12_O_6_), arabinose (C_5_H_10_O_5_), and glycerol (C_3_H_8_O_3_)—on BC production by *Komagataeibacter hansenii*. The varying molecular weights and structural characteristics of these carbon sources provide a framework for examining their influence on BC yield, fiber morphology, and network properties. BC production was monitored through daily measurements of optical density and pH levels in the fermentation media from day 1 to day 14, providing valuable insights into bacterial growth kinetics and cellulose synthesis rates. Scanning electron microscopy (SEM) was used to elucidate fibril diameter and pore size distribution. Wide-angle X-ray scattering (WAXS) provided a detailed assessment of crystallinity. Selected BC pellicles were further processed via freeze-drying to produce a foam-like material that maximally preserves the natural three-dimensional structure of BC, facilitating the incorporation and release of lidocaine hydrochloride (5%), a widely used local anesthetic. The lidocaine-loaded BC foams exhibited a sustained and controlled release profile over 14 days in simulated body fluid, highlighting the importance of the role of carbon source selection in shaping the BC network architecture and its impact on drug release profile. These results highlight the versatility and sustainability of BC as a platform for wound healing and drug delivery applications. The tunable properties of BC networks provide opportunities for optimizing therapeutic delivery and improving wound care outcomes, positioning BC as an effective material for enhanced wound management strategies.

## 1. Introduction

Bacterial cellulose (BC) is a biopolymer of growing interest due to its unique combination of structural and mechanical properties, alongside its potential for sustainable applications [1]. BC is composed of highly crystalline nanofibrils, with individual fibrils measuring between 6–10 nm in cross-sectional thickness, which bundle into larger fibers, typically 30–80 nm wide, forming a three-dimensional (3D) network [2]. These nanofibrils are deposited in successive layers, creating a highly organized, multilayered structure that enhances both the mechanical strength and flexibility of the material. The precise size and arrangement of the fibrils depend on the cellulose-producing organism, contributing to BC’s mechanical durability [3]. This hierarchical, layered architecture is advantageous for biomedical applications, such as wound healing and drug delivery, where the material’s mechanical properties and porosity play a critical role in its performance [2,4,5,6].

BC is synthesized extracellularly by various bacteria, including *Komagataeibacter hansenii*, producing naturally a material of high purity, thus avoiding the need for complex chemical extraction processes. BC formation increases proportionally with the fermentation time and is driven by C-H bonding during the biosynthesis process. As the pellicle growth rate diminishes and the bacteria become fully entrapped within the cellulose matrix, the production of BC approaches its maximum limit [7]. This biosynthesis of BC can be finely tuned by altering the carbon sources in the growth medium, significantly affecting the material’s structural and functional properties [3,8]. Sugars such as raffinose (C_18_H_32_O_16_), sucrose (C_12_H_22_O_11_), glucose (C_6_H_12_O_6_), and arabinose (C_5_H_10_O_5_), as well as glycerol (C_3_H_8_O_3_), can serve as carbon sources for bacterial growth, and their molecular structure and molecular weight can affect the BC production yield, network formation, and fiber arrangement [9,10]. These variations in carbon sources impact the mesoscale assembly of the BC network, influencing its fiber organization, mechanical properties, porosity, and overall material functionality [8].

Studies have shown that glycerol leads to the highest BC production compared to glucose and more complex sugars, with superior metabolic efficiency in terms of BC yield per g/mol of carbon consumed. Additionally, BC produced with glycerol demonstrated higher crystallinity than glucose-based BC, suggesting that simpler carbon sources are metabolized more efficiently [11]. Carbon source selection also significantly influences BC productivity and structure, with sucrose achieving yields comparable to glucose, particularly when fermentation times exceed 96 h [12]. Another study found that while glucose produced the largest pore diameters and had the highest yield, sucrose resulted in the largest fibril diameters, with only slightly lower yields than glucose, further emphasizing the role of carbon sources in shaping both BC morphology and productivity [13].

The ability to modify BC through functionalization, primarily due to the abundant hydroxyl groups along its polymer chains, further enhances its versatility [14]. BC has been functionalized for applications in drug delivery, antimicrobial systems, and tissue engineering, with modifications often aimed at improving mechanical strength, adding new bioactive properties, or tailoring its degradation profile [4,15,16]. In static cultures, BC forms a pellicle at the liquid–air interface, where the growth conditions, including the surface-to-volume ratio, play a critical role in determining the final structure of the material. Higher ratios, for example, increase oxygen availability, which is essential for cellulose production, leading to more efficient synthesis. The resulting pellicle exhibits a multi-layered architecture, characterized by interwoven, entangled fibers that create a mechanically strong network across the layers [8].

In biomedical applications, BC presents a sustainable alternative to traditional petroleum-derived polymers, which are non-biodegradable and contribute to environmental pollution and hazardous waste accumulation [2]. Its biocompatibility, biodegradability, and porous structure make BC well-suited for wound healing, where it can effectively absorb exudates, conform to wound surfaces, and serve as a protective barrier against infections [17,18]. Additionally, BC’s chemical inertness, high purity, and mechanical strength enhance its utility in promoting epithelialization in burn treatments, including severe third-degree burns [18,19,20]. BC has also proven to be an efficient drug delivery carrier for the incorporation and controlled release of lidocaine, a commonly utilized pain-relieving agent in clinical settings. [4,5]. When applied as a wound dressing, BC’s distinctive matrix structure and its capacity to reduce fibrotic adhesion make it an advantageous material for a wide range of biomedical applications [21].

Lidocaine is a widely used topical anesthetic, particularly in the management of burn injuries, where effective pain control is critical. Case studies suggest that lidocaine can enhance analgesic efficacy, reduce the negative effects of opioid administration, and lessen the need for increasing opioid doses in burn patients [17,19]. In addition to providing localized relief, lidocaine minimizes systemic side effects, making it a preferred option for pain management in wound care [19,22]. The incorporation of lidocaine into advanced wound dressings enables a controlled and sustained release of the drug, ensuring consistent pain relief over extended periods. This gradual release mechanism helps maintain therapeutic drug levels while minimizing the risk of toxicity associated with higher doses [20]. Additionally, as BC membranes can be tailored into various shapes and sizes, this makes them particularly effective for covering large or irregular wound areas. BC-based scaffolds loaded with lidocaine not only preserve the stability of the drug but also optimize its release rate, providing an effective and balanced approach to both pain management and wound healing, especially in burn care [5].

This study investigates how different carbon sources influence the structure, mechanical properties, and biomedical potential of BC, with a particular emphasis on its use in wound management systems. By examining the relationship between carbon source selection, BC network formation, and functional performance, this research aims to advance the development of BC-based materials for sustainable, high-performance biomedical applications, such as tissue engineering, drug delivery, and biosensing.

## 2. Materials and Methods

### 2.1. Culture Medium and Fermentation Conditions

*Komagataeibacter* hansenii (ATCC 53582) was used for BC production, with the culture maintained on Hestrin–Schramm (HS) agar medium. The standard HS medium contained (%, *w*/*v*): 2% glucose, 0.5% peptone, 0.5% yeast extract, 0.27% Na_2_HPO_4_, and 0.115% citric acid, following the formulation described by Hestrin and Schramm [23]. To standardize bacterial growth, pre-inoculum cultures were prepared by incubating the bacteria statically at 30 °C for 48 h. The resulting culture suspension was measured for optical density (OD) at 600 nm using a microplate reader (Agilent Biotek, Santa Clara, CA, USA), and the OD600 was adjusted to 0.3 (~10^6^ CFU/mL) to ensure consistent inoculation across all experiments.

**Figure 1:**
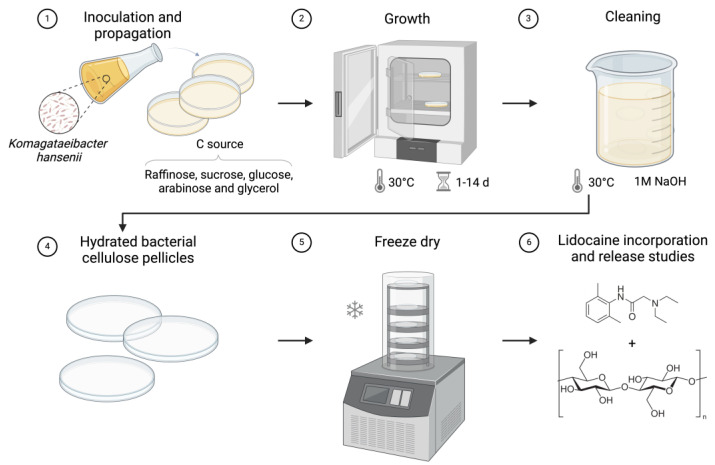
Schematic of bacterial cellulose cultivation and sample preparation.

To investigate the effects of different carbon sources on BC production, the bacterium was cultured using standard and modified HS media where the standard glucose (GLU) was replaced with raffinose (RAF), sucrose (SUC), arabinose (ARA), and glycerol (GLY). The carbon content for each alternative sugar was calculated based on their molecular weight to maintain an equivalent carbon concentration to that of the original HS medium containing glucose. The calculated concentrations for each sugar were as follows: 18.67 g/L of raffinose, 19.23 g/L of sucrose, 19.96 g/L of arabinose, and 20.45 g/L of glycerol. All cultures were grown under static conditions at 30 °C for 14 days to promote BC pellicle formation at the air–medium interface (Figure 1). All experiments were conducted in triplicate.

### 2.2. Measurements of Media Optical Density and Cellulose Yields

From day 1 to day 14, the pH and wet yield of the cultures were measured daily. A simple assay using optical density (OD) measurements was employed to assess the extent of bacterial growth in each sample. The pre-inoculum used to inoculate all cultures had the same OD to ensure consistency and precision in the assay. A baseline measurement was taken using the pre-inoculum in the liquid medium containing the respective carbon sources. The liquid medium from each time point was preserved and analyzed with a microplate reader (Agilent Biotek, Santa Clara, CA, USA) to determine OD at 600 nm for all carbon sources, establishing growth curves of *K. hansenii* and providing insights into bacterial proliferation throughout the incubation period.

Post-incubation, BC pellicles were harvested, rinsed with deionized water, and gently blotted with a kimwipe to remove surface water before their wet weight (W_wet_) was recorded. Selected pellicles were then allowed to dry at room temperature until reaching a constant weight at which point the dry weight (W_dry_) was recorded.

The water holding capacity (WHC) was calculated using the formula [24]:

WHC = [(W_wet_ − W_dry_)/W_dry_] × 100 (1)

Following these measurements, the cellulose pellicles were purified using 1 M NaOH and washed repeatedly until a neutral pH was reached. Post-purification, the pellicles underwent further processing, including freeze-drying, before the incorporation of lidocaine, as demonstrated in Figure 1.

### 2.3. Structural Analysis

Four specific timepoints were selected for all carbon sources corresponding to the transition from the exponential growth phase to the stationary phase of the *K. hansenii* proliferation. These selected timepoints were utilized for conducting characterizations to assess the structural properties of the BC pellicles produced under various growth conditions at different length scales.

#### 2.3.1. Sample Preparation

Cleaned BC pellicles were prepared for characterizations following one of two methods. Method one places BC pellicle between absorbent mats under weights to remove water from the network, creating a densified BC membrane. Method two uses a Labconco FreeZone 2.5 Plus 2.5 Liter Cascade Benchtop Freeze Dry System (Labconco Corporation, Kansas City, MO, USA), following the protocols established by Paakkonen et al. [25] to produce freeze-dried BC with a foam-like appearance where its intrinsic fiber network structure is preserved. Throughout the rest of the manuscript, BC samples produced using these two methods will be referred to as press-dried BC (PD BC) and freeze-dried BC (FD BC).

#### 2.3.2. Scanning Electron Microscopy (SEM)

All samples were coated with a 4 nm layer of platinum using an EM ACE600 sputter coater (Leica Microsystems GmbH, Wetzlar, Germany) prior to imaging. PD BC membranes were used for surface imaging, while FD BC membranes were used for cross-section imaging. Subsequent imaging was performed on an Apreo VP SEM (Thermo Fisher Scientific, Waltham, MA, USA) in optiplan mode using an accelerating voltage of 2 kV and a current of 10 pA. Surface images of PD BC were analyzed using ImageJ software (Version 1.54j), National Institutes of Health, Bethesda, MD, USA), with 100 measurements taken from a single technical replicate. Fiber diameter distribution was determined to closely approximate the true values, with priority given to non-overlapping regions to enhance the accuracy of the measurements. Cross section images of FD BC were also analyzed to measure the approximate pore size in the freeze-dried BC structure. The same number of measurements was taken as in the surface image analysis.

#### 2.3.3. Wide-Angle X-Ray Scattering (WAXS)

WAXS scans of the press-dried BC membranes were acquired using a Xenocs Xeuss 3.0 system (Xenocs SAS, Sassenage, France), equipped with a CuKα radiation source providing a wavelength of 1.54 Å. Measurements were carried out for a duration of 120 s or 180 s depending on sample thickness in standard mode, triplicate scans were conducted on each sample at locations spaced at least 1.5 mm apart. Azimuthal averaging was employed to convert the 2D WAXS scattering patterns into 1D profiles using the Xenocs XSACT software (Xeuss 3.0 platform).

The crystallinity index (CI), an indication of the ratio of the crystalline phase and the amorphous phase within the elementary cellulose fibrils, was calculated using the method introduced by Segal et al. by taking the ratio between the intensity of the (110) peak (I_110_) and that of the amorphous baseline (I_am_, measured at approximately 2θ = 18.5°, θ being the Bragg angle) following Equation (2) [26].

Sizes of crystalline regions/crystallites in the elementary BC fibrils, or coherent scattering regions (CSR) were calculated from the WAXS spectra as well using the Scherrer equation (Equation (3)) [27], where D_hkl_ is size of the crystallite in the direction perpendicular to the lattice plane represented by Miller indices hkl, K is the crystal shape dependent constant (0.94), λ is the X-ray wavelength, and β_hkl_ is the line broadening at half the maximum intensity (FWHM) calculated at the corresponding diffraction peak. Specifically, the CSR size calculated from the (11$\bar{4}$) diffraction peak corresponds to the crystallite dimension along the longitudinal direction of elementary BC fibrils, while CSR sizes calculated from the (100), (010), and (110) peaks describe the crystallite profile along the cross-section of the elementary BC fibrils [28,29].

CI = [(I_110_ − I_am_)/I_110_] × 100 (2)

D_hkl_ = (K × λ)/(β_hkl_ × cos(θ)) (3)

#### 2.3.4. Fourier Transform Infrared Spectroscopy (FTIR)

The PS BC samples from the selected timepoints were analyzed using a ThermoNicolet iS10 FT-IR spectrometer (Thermo Fisher, Waltham, MA, USA), with spectra recorded in the range of 4000 to 400 cm^−1^, utilizing 20 scans and a resolution of 4 cm^−1^.

### 2.4. Drug Delivery Application

Three samples from distinct carbon sources were selected based on their cellulose content. These samples were subjected to an FD process using the Labconco FreeZone 2.5 Plus 2.5 Liter Cascade Benchtop Freeze Dry System (Labconco Corporation, Kansas City, MO, USA). The pellicles were not pre-frozen prior to the FD procedure, in order to preserve their intrinsic three-dimensional network, hypothesized to enhance drug uptake capacity [25].

#### 2.4.1. Preparation of Lidocaine-Loaded BC Dressings

A 5% lidocaine hydrochloride solution (Cristália, Itapira, Brazil) was used as the therapeutic agent. The FD pellicles were immersed in this solution, with the amount of solution calculated to achieve an approximate lidocaine loading of 50 mg per pellicle, based on their wet weight after drying. The samples were maintained on an orbital shaker (Incushaker 10L, Benchmark, Sayreville, NJ, USA) set to 80 rpm for 6 h at 30 °C, ensuring optimal penetration of the lidocaine into the pellicles, promoting uniform drug distribution within the matrices. Drug loading was determined gravimetrically by calculating the difference in mass between the dry BC pellicles and the lidocaine-loaded pellicles. The drug loading for each sample was calculated based on the weight difference.

#### 2.4.2. In Vitro Lidocaine Release

The freeze-dried, lidocaine-loaded BC pellicles were immersed in 15 mL of Earle’s Balanced Salts Solution (EBSS) (Sigma Aldrich, St. Louis, MO, USA), used as a simulated body fluid (SBF) to mimic physiological conditions (pH of 7.4). The composition of EBSS closely replicates extracellular fluid, enabling the assessment of drug release, tissue interaction, and wound healing kinetics in a controlled in vitro environment, providing an accurate reflection of the material’s performance in potential in vivo applications [30]. The samples were subjected to constant shaking at 120 rpm at 37 °C to simulate physiological movement. At specific time intervals (1, 2, 3, up to 14 days), 1.5 mL of the release medium was sampled, followed by the addition of fresh SBF to maintain sink conditions and ensure consistency throughout the experiment. Lidocaine concentrations in the release medium were measured via absorbance at 263 nm using a microplate reader (Agilent Biotek, Santa Clara, CA, USA) [31]. The cumulative release percentage was calculated by normalizing the amount of lidocaine released at each time point to the initial drug loading, which was determined gravimetrically. The total amount of lidocaine released over time was expressed as a percentage of the initial loaded amount, providing insights into the drug release profile of the BC matrixes.

#### 2.4.3. Swelling Capacity and Cyclic Testing

To evaluate the volume swelling properties and recyclability of FD and PD BC samples, glycerol-derived BC pellicles from 9-day cultures were selected based on their superior lidocaine release performance. The FD pellicles were processed without prior freezing.

The swelling capacity was determined as the ratio of wet mass to dry mass, expressed as a percentage, in accordance with Equation (4). Measurements were assessed over three cycles, with each test conducted in triplicate. The samples were immersed in deionized water for 24 h to achieve full swelling. After immersion, the samples were gently blotted to remove excess water and weighed to determine their swollen mass. The samples were then air-dried until constant weight was reached, completing one cycle. This process was repeated for a total of three cycles. Swelling capacity was calculated as the ratio of wet mass to dry mass, expressed as a percentage, with the first cycle’s value normalized to 100%. The results, along with standard deviations, were analyzed to evaluate the maintenance of swelling capacity over multiple cycles.

Swelling capacity = [(Wet mass − Dry mass)/(Dry mass)] × 100(4)

## 3. Results and Discussion

### 3.1. Effect of Carbon Source

#### 3.1.1. Bacterial Growth

The choice of nutrients in the growth medium plays a critical role in determining both the yield and the characteristics of BC produced by bacteria such as *K. hansenii* [9,14,32]. Given the high cost of glucose, which is a standard sugar for BC production, alternative carbon sources have been investigated to improve the cost-efficiency of the production yield. These alternatives not only reduce production costs but also result in variations in the BC’s structural properties, such as fiber arrangement, crystallinity, WHC and porosity [14,32,33], highlighting the importance of selecting the appropriate carbon source to tailor BC for specific functional applications.

**Figure 2:**
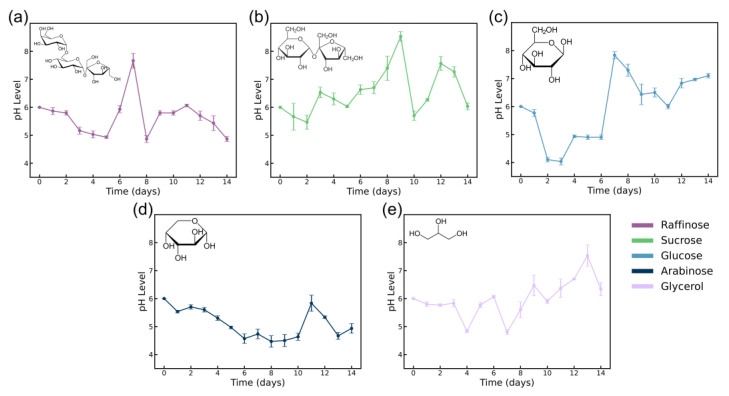
pH progression in growth media during BC production with Raffinose media (**a**), Sucrose media (**b**), Glucose media/standard HS media (**c**), Arabinose media (**d**), and Glycerol media (**e**).

**Figure 3:**
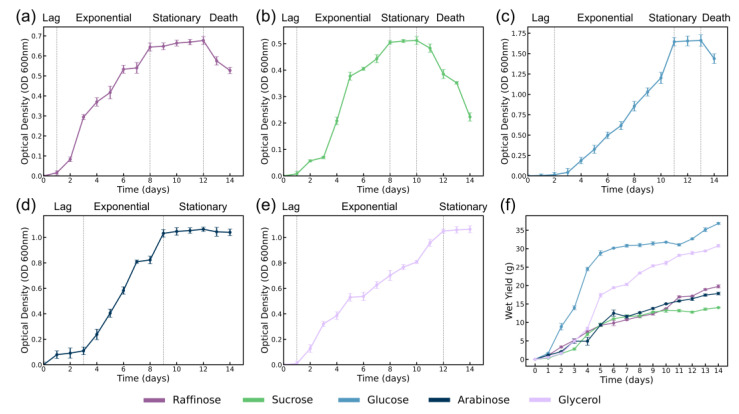
Bacteria growth curves from optical density measurements in Raffinose media (**a**), Sucrose media (**b**), Glucose media/standard HS media (**c**), Arabinose media (**d**), Glycerol media (**e**), and BC wet yield at different time points for all media types (**f**).

In this study, raffinose, sucrose, glucose, arabinose, and glycerol were compared to assess their impact on the growth of *Komagataeibacter hansenii* (ATCC 53582) and its BC production. To better understand the metabolic processes and their influence on BC synthesis, the pH of the culture medium was monitored throughout the 14-day fermentation period. The results demonstrated in Figure 2 reveal distinct pH trends corresponding to the specific carbon sources used, which reflect the underlying metabolic pathways and their byproducts, which is further supported by the OD data in Figure 3a–e.

Raffinose, a trisaccharide composed of galactose, glucose, and fructose, undergoes a two-step enzymatic hydrolysis during bacterial fermentation, where galactose is released by α-galactosidase, followed by the hydrolysis of sucrose into glucose and fructose [34]. This sequential breakdown occurs over time and significantly affects BC synthesis, explaining the slow initial drop in pH (Figure 2a). Around day 6, a sharp rise in pH is observed, likely due to the complete hydrolysis of raffinose, leading to the rapid metabolism of glucose and fructose [34,35]. Raffinose is less commonly used in BC production compared to simpler sugars like glucose, but studies have shown that it can provide a steady supply of carbon over a longer fermentation period, influencing bacterial growth kinetics [35,36]. This is further supported by a noticeable spike in pH around day 7, indicating a complex metabolic shift as the bacteria is expected to transition from raffinose hydrolysis to the metabolism of its constituent sugars. As shown in Figure 3a, during the stationary phase (days 8–12), gluconic acid production leads to a subsequent pH drop as metabolic byproducts accumulate. The eventual transition to the death phase is marked by a further decrease in pH due to nutrient depletion and continued acid production [37]. The bacterial growth curve mirrors these pH changes, with a delayed lag phase, exponential growth between days 4 and 10, and a stable stationary phase thereafter.

Sucrose, a disaccharide composed of glucose and fructose, serves as an efficient carbon source for *K. hansenii* growth and eventual BC production through its enzymatic hydrolysis [38]. This hydrolysis breaks down sucrose into glucose and fructose, which directly enter central metabolic pathways like glycolysis and the pentose phosphate pathway, facilitating energy production for bacterial proliferation and cellulose biosynthesis [12]. The pH profile during sucrose fermentation indicates a series of metabolic adjustments (Figure 2b). A sharp rise in pH after day 7 suggests a buffering effect in the medium or a temporary reduction in the accumulation of acidic by-products, such as gluconic acid, which are typically associated with glucose metabolism [9,37]. This trend aligns with metabolic shifts of *K. hansenii*’s glucose metabolism, where gluconate is a key intermediate in the oxidative pathway [37]. Post-day 10, the pH drops again, coinciding with the onset of the stationary or death phase. This decline is likely due to the accumulation of gluconic acid, which is a byproduct of glucose oxidation, that has been observed to lower pH during late fermentation stages [39,40]. This drop aligns with the stationary phase and nutrient depletion, signaling the beginning of the death phase (Figure 3b). The bacterial growth curve reflects these dynamics, with rapid initial growth tapering off into a plateau after day 10, consistent with the earlier nutrient depletion compared to raffinose.

As anticipated, glucose, being a monosaccharide and the direct precursor for cellulose synthesis, resulted in the highest BC production among the studied carbon sources [14]. The growth curve demonstrates a well-defined exponential phase from day 2 to day 10. The rapid depletion of glucose during early fermentation stages is correlated with a significant decrease in pH at the early stages of the fermentation, driven by the production of acidic metabolic by-products like gluconic acid [1]. This pronounced growth is likely due to the direct metabolic entry of glucose into central pathways such as glycolysis, providing an immediate and efficient energy source for BC biosynthesis [1,38]. However, the slight increase in pH observed after day 6 suggests metabolic adjustments within the bacterial culture, possibly through the utilization of accumulated acids or changes in the rate of by-product formation. This temporary stabilization could reflect a shift in bacterial metabolism aimed at maintaining a balanced environment despite nutrient depletion [38]. The pH drops sharply again after day 10, which could be correlated with glucose depletion and the transition to the death phase. The bacterial growth curve highlights this trend, with a steep rise in OD600 during the exponential phase and a sharp decline as glucose is exhausted, reflecting the carbon source’s immediate availability and metabolic efficiency (Figure 3c).

Arabinose, a pentose sugar metabolized by bacteria through the pentose phosphate pathway [41,42], demonstrated a relatively stable pH profile throughout fermentation, compared to other carbon sources (Figure 2d). This characteristic suggests a slower, more controlled metabolic process, as arabinose does not generate significant amounts of acidic by-products like gluconic or acetic acid, which is often produced in larger quantities with glucose or sucrose fermentation. The absence of rapid pH decline correlates directly with the bacterial growth curve, which exhibits a consistent rise followed by a prolonged stationary phase starting around day 8, without entering a clear death phase (Figure 3d), supporting the idea that arabinose metabolism avoids the rapid nutrient depletion and toxic by-product accumulation that disrupt cellular processes in later stages [10,42]. The pH results during this period supports the hypothesis of a slower metabolic process [11].

Glycerol, a versatile three-carbon molecule, demonstrates unique characteristics in BC production, particularly through its metabolic pathways. In the case of *K. hansenii*, glycerol is processed via the glycerol-3-phosphate pathway, feeding into central metabolic routes like the tricarboxylic acid cycle, which enables a steady flux of energy without rapid acid accumulation [43], shown with a steady pH until day 3 (Figure 2e). However, in this study, glycerol led to significant pH oscillations throughout the fermentation process after day 4, challenging the conventional understanding of its stabilizing role. Our data indicate complex metabolic dynamics at play (Figure 2e), which could be attributed to varying levels of acid or byproduct production at different stages of the growth cycle. While previous studies suggested that glycerol minimizes the formation of acidic byproducts such as gluconic acid [43,44], the observed pH behavior in this work suggests more complex metabolic pathways. The mid-fermentation pH rises likely point to the consumption of acidic intermediates, whereas subsequent drops may be due to renewed acid production from incomplete glycerol breakdown, particularly under nutrient-limiting conditions.

Each carbon source uniquely influences the BC production process, affecting not only the yield but also bacterial growth dynamics, metabolic by-products, and pH variations. Carbon sources like raffinose, glucose, and sucrose exhibited notable pH fluctuations, primarily due to the accumulation and subsequent consumption of gluconic acid during glucose oxidation [40]. A non-monotonic pH profile in BC production has been reported before, where these pH changes were attributed to the complexity of reactions occurring in the media, where the formation and consumption of gluconic acids reflect dynamic metabolic activity [44]. In contrast, arabinose and glycerol fostered more stable growth conditions with less pronounced fluctuations in pH, which corresponded to an extended stationary phase and delayed onset of the death phase (Figure 3d,e). This stability in metabolism suggests a more controlled fermentation process. These findings emphasize the importance of carbon source selection in optimizing BC synthesis, offering the potential for fine-tuning the structural and functional properties of BC for diverse biomedical and industrial applications.

#### 3.1.2. Bacterial Cellulose Production

The OD measurements presented in Figure 3a–e demonstrate the distinct bacterial growth patterns observed in media containing different carbon sources. These growth curves provide insight into the metabolic activity of *K. hansenii* and how it is influenced by the carbon source. The wet yield data in Figure 3f illustrate distinct trends in BC production across different carbon sources, with glucose and glycerol showing the highest yields compared to raffinose, sucrose, and arabinose. This is consistent with the known efficiency of simpler sugars in supporting BC production [8,39,45].

Both glucose and glycerol demonstrated significantly higher BC yields compared to other carbon sources, reflecting their metabolic efficiency. Glucose, being a fundamental substrate for bacterial metabolism, supports the most efficient BC production due to its immediate availability for glycolysis and other key biosynthetic pathways. The sharp increase in wet yield during the exponential growth phase (Figure 3c) aligns with its efficient metabolism through the glycolytic pathway, providing immediate energy and metabolic intermediates necessary for BC synthesis [46]. Glucose also exhibits a well-defined stationary phase from day 10, during which BC production starts to stabilize, reflecting the depletion of nutrients and reduced bacterial activity. Similarly, glycerol demonstrates significant potential as a carbon source, resulting in consistently high BC yields throughout the fermentation process (Figure 3e). Although slightly less efficient than glucose, glycerol’s slower metabolic rate, as reflected in its steadier growth curve, supports prolonged bacterial growth and BC production.

Sucrose, a disaccharide, also requires initial hydrolysis by invertase to yield glucose and fructose, which are then metabolized through glycolysis [9,38]. Despite being a simpler sugar than raffinose, sucrose showed a gradual increase in BC yield but remained significantly lower than glucose and glycerol. The pH curve for sucrose cultures revealed a sharp increase around day 8, which likely indicates a shift in bacterial metabolism, followed by a rapid drop in pH, potentially due to the accumulation of acidic byproducts during the fermentation process. Despite these fluctuations, BC production remained lower than glucose and glycerol, suggesting that sucrose is less efficient as a carbon source for *K. hansenii*.

Arabinose, although a monosaccharide, demonstrated BC yields comparable to the more complex sugar, raffinose. That can be attributed to differences in their metabolic pathways rather than molecular complexity alone. Arabinose, entering through the pentose phosphate pathway, requires the synthesis and activation of specific enzymes, leading to a longer lag phase [42]. This slower initiation delays BC synthesis, reflected in the extended stationary phase without a clear death phase, as seen in Figure 3d,f. Arabinose’s stable pH and a slower rate of acidic byproduct formation likely contribute to sustained bacterial activity over time, enabling steady but moderate BC production.

Raffinose, despite being a trisaccharide, is metabolized more efficiently once enzymatically hydrolyzed into glucose and fructose. These monosaccharides can then rapidly enter the glycolytic pathway, accounting for raffinose’s shorter lag phase in contrast to arabinose (Figure 3a,d). However, due to the additional enzymatic steps involved in raffinose metabolism, its exponential growth phase is prolonged, ultimately leading to a similar BC yield curve as arabinose [35]. Despite this, raffinose offers a steady carbon supply during fermentation, particularly in the later stages, allowing for more prolonged BC production. The delayed onset of BC production for arabinose, paired with a more prolonged stationary phase, contrasts with raffinose’s quicker initial response but a lengthier exponential phase, balancing their overall yields.

The carbon source has a pronounced effect on BC production, with glucose and glycerol providing higher yields due to their more direct metabolic pathways. In contrast, raffinose and sucrose, which require enzymatic hydrolysis, show delayed and less efficient BC production. The stability of arabinose, despite lower yields, underscores its potential in applications requiring extended fermentation times. These findings highlight the need for careful selection of carbon sources depending on the desired characteristics and yield of the BC material.

#### 3.1.3. Structural Analysis

The samples collected between days 9 to 12 were chosen for further characterization due to their relevance in the transition from the exponential phase to the stationary phase of bacterial growth, where the rate of cellulose production begins to plateau and stabilize. At this stage, the bacterial biomass and metabolic activities are relatively consistent for subsequent analysis. The uniformity of the pellicles, as indicated by consistent wet yields during this period makes this window ideal for evaluating key material properties such as WHC, fiber network structure, and crystallinity.

**Figure 4:**
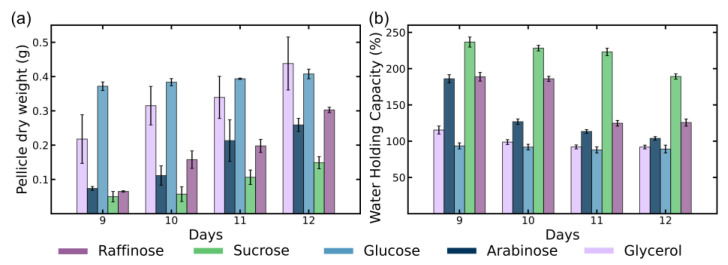
Dry weight (**a**) and water holding capacity (WHC) (**b**) of BC pellicles of BC membranes produced in different growth media.

Between days 9 and 12, all carbon sources demonstrate a general increase in dry weight, suggesting the continued production of BC during the stationary phase (Figure 4a). Although bacterial metabolic activity slows during this period, cellulose biosynthesis persists, likely facilitating the ongoing bundling and densification of BC fibers within the network. This observation is consistent with earlier studies that report cellulose accumulation during the stationary phase despite reduced cellular proliferation [21,43].

During the stationary phase, the BC fibers continue to bundle, leading to a more tightly packed structure at the mesoscale [12]. This densification is reflected in the declining WHC across most carbon sources from day 9 to day 12 as shown in Figure 4b. As the cellulose network matures and compacts, its ability to retain water diminishes [12,39]. Notably, both glucose and glycerol exhibited consistently low WHC values, indicating that the BC networks produced with these carbon sources have higher fiber content and reduced water retention capacity compared to BC grown using the other sugars.

Despite its lower dry weight, sucrose maintained a high WHC compared to all other sugar options. This reflects the delay in network formation, as seen in the growth data. It also could indicate that the sucrose-fed bacterial cultures had formed BC pellicles with a more open, porous network structure that allowed for high water retention. Raffinose-grown BC showed high initial WHC and a marked decline by day 12. The delayed hydrolysis of raffinose into simpler sugars could explain this trend, with a subsequent acceleration in cellulose production leading to a more compact network structure [35]. Arabinose-grown BC also exhibited a similar decrease in WHC over the time period tested. From Figure 3a,d, we can see that both arabinose and raffinose cultures are in the steady stage of BC growth, which likely suggests a change in network assembly mechanism from a volumetric expansion mode in the exponential stage towards a network densification mode in the steady stage.

**Figure 5:**
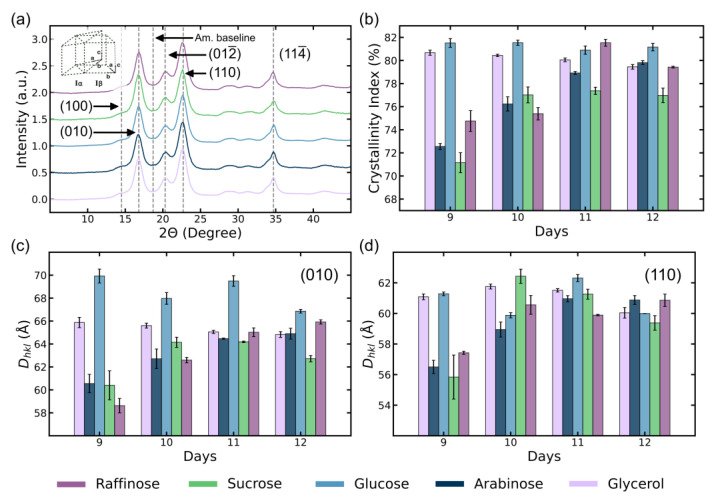
One-dimensional radial WAXS profile (azimuthal average) at 12 D growth time (**a**), calculated crystallinity index evolution (**b**), dimensions of coherent scattering regions (CSR) calculated from WAXS profiles in (010) direction (**c**), and (110) direction (**d**) of BC membranes produced in different growth media.

From the WAXS profiles of the BC membranes (Figure 5a and Figure S1), we can obtain information regarding the crystallinity of the cellulose that makes up the network and other information such as the type of cellulose crystal (crystal system) and crystallite dimensions. From the peak locations and intensity profiles of the WAXS spectra, we can confirm the dominant form of cellulose crystal present in the BC grown using all types of sugars is I_α_, which is in good agreement with the literature [28,47]. As opposed to the monoclinic cellulose I_β_ that is more commonly seen in the cell wall of plants, microbially synthesized cellulose is typically found to contain a high percentage of the triclinic cellulose I_α_ [48]. The Bragg diffraction peaks in the WAXS spectra at 2θ angles of 16.8°, 20.3°, 22.6°, and 34.7° correspond to the (100), (010), (01$\bar{2}$), (110), and (11$\bar{4}$) crystal planes are in good agreement with values reported in prior literature [28,49].

The time-evolution curves of the CI for BC grown using different sugars are shown in Figure 5b. We see BC grown using glucose and glycerol feedstock having the highest average CI at above 80%, which stayed relatively constant across the time period tested. We also see an increasing trend in the CI of BC grown using arabinose, sucrose and raffinose media across the same time period, going from 72.6%, 71.1% at day 9, and 74.8% to 79.8%, 77.0%, and 79.4% at day 12, respectively. Interestingly, this period coincides largely with the exponential phase of glucose and glycerol cultures, and the stationary/death phase of the arabinose, sucrose, and raffinose cultures.

The calculated values of CSR dimensions of BC produced using different sugars are tabulated in Table S1. The cross-sectional dimensions of BC crystallites we calculated are in good agreement with the diameter of elementary BC fibrils in prior literature measured through transmission electron microscopy (TEM) and atomic force microscopy (AFM) [29,50]. CSR dimensions in the (010) and (110) directions are plotted with respect to growth time in Figure 5c,d. We can see the BC grown using glucose and glycerol tend to have the largest crystallite dimensions in the (010) and (110) directions (65.9 Å and 61.1 Å for GLY9, 69.9 Å and 61.3 Å for GLU9, respectively), which implies larger elemental fibril diameters compared to BC grown using other sugars (with $D_{010}$ from 58.6–60.6 Å and $D_{110}$ from 55.8–57.4 Å, respectively). This trend can be positively correlated with total BC production, which as previously discussed, is highest for glucose and glycerol-based media. We also observe a general increasing trend in (010) and (110) CSR dimensions for BC grown using arabinose and raffinose, indicating an increase in elemental fibril diameters with growth time in the period studied. For sucrose-grown BC, we see an interesting bell-shaped curve in calculated CSR dimensions in (010) and (110), which coincides with the portion of its bacterial growth curve transitioning from stationary to death phase.

While the FTIR spectra for BC films grown over time using different carbon sources appear largely similar, minor variations were noted, particularly in the O-H stretching region and around 1733 cm^−1^, as detailed in Figure S2 and Table S2. These variations could be attributed to slight differences in crystallinity, which was a factor also observed in our WAXS data. However, no discernible trends were identified across the samples; hence, no further detailed investigation was conducted on this aspect.

### 3.2. Drug Loading and Release Efficiency in BC Foams

#### 3.2.1. In Vitro Lidocaine Release

The samples GLY 9 (glycerol, 9 days), ARA 12 (arabinose, 12 days), and RAF 11 (raffinose, 11 days) were chosen for the in vitro lidocaine release studies due to their comparable crystallinity, similar thicknesses (measured with a microcaliper), optimal growth stages, and consistent fiber networks. The comparable crystallinity across the samples ensured that variations in drug release could be attributed to the fiber network properties, providing a reliable basis for studying sustained lidocaine diffusion.

The lidocaine incorporation for each sample was controlled and calculated gravimetrically (see methods). The wet weights of the pellicles were 2.374 g ± 0.078 for GLY, 2.442 g ± 0.089 for ARA, and 2.401 g ± 0.091 for RAF. This corresponds to lidocaine incorporations of approximately 47.48 mg ± 1.56 mg for GLY, 48.84 mg ± 1.48 mg for ARA, and 48.82 mg ± 1.62 mg for RAF, allowing direct comparisons of the drug release profiles across the different BC matrices.

**Figure 6:**
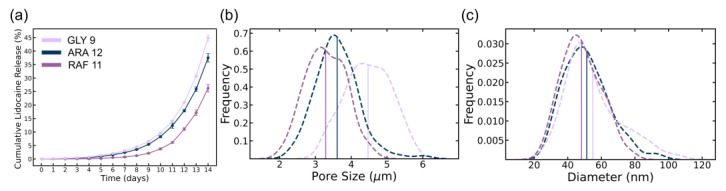
Cumulative Lidocaine release (**a**), pore size (**b**), and fiber diameter (**c**) of GLY 9, ARA 12, and RAF 11 BC.

Pore sizes measured from SEM images of the three samples used in the lidocaine release study are shown in Figure 6b. We can see the GLY 9 has the largest average pore size after freeze-drying followed by the ARA 12 and the RAF 11. Similarly, the fiber diameter in the three different samples follows the same trend of d_GLY9_ > d_ARA12_ > d_RAF11_ (Figure 6c). The fiber diameter together with pore size provides a qualitative understanding of the network structure where it can be concluded that GLY 9 has a “looser” network structure compared to ARA 12 and RAF 11.

The cumulative lidocaine release profiles obtained by gravimetric analysis (Figure 6a) reveal the distinct influence of the carbon source and their fiber network structures on BC’s drug release profile, becoming especially pronounced after day 4. Glycerol-based BC, with a cumulative release exceeding 45% by day 14, benefits from its larger pore size and, as a result, a more permeable structure. This steady, sustained diffusion of lidocaine makes it ideal for long-term pain management, ensuring therapeutic levels are consistently maintained throughout the treatment period. This continuous release not only reduces the frequency of reapplication but also enhances patient compliance, an important factor in the effective management of chronic wounds [51].

Similarly, raffinose-based BC, despite a lower cumulative release (~27% by day 14), demonstrates effective long-term drug delivery. Its fiber network provided a more controlled and gradual release of lidocaine, making it well-suited for chronic wound care where a slower, steady release is beneficial. By avoiding rapid bursts of drug release, raffinose BC helps mitigate potential side effects while ensuring prolonged analgesic effects, making it particularly useful for conditions requiring extended treatment, such as diabetic ulcers or pressure sores [51,52].

The lidocaine release profiles from all samples exhibit an exponential release profile, characterized by a continuous acceleration of release [53], indicative of sustained drug diffusion through the BC matrix over the entire 14-day period. Our results indicate that the BC structure, influenced by different carbon sources, plays a crucial role in controlling the lidocaine release profile. The sustained release observed over 14 days in our study contrasts with other studies using BC matrices with different structural properties, which have shown either faster release rates (over 90% within 20 min) or lower cumulative percentages of lidocaine release from BC composites [54,55]. This highlights the potential of modulating the carbon source to modulate the drug release profile for specific biomedical applications. The three carbon sources follow a pattern where drug release accelerates after an initial lag phase, becomes more rapid during the exponential phase, and plateaus as equilibrium is reached. The lidocaine release profile shows a clear correlation between the BC matrix pore size/fiber diameter and release rate. In this system, where the drug release is diffusion-limited, a network with smaller pores restricts diffusion pathways and thus results in a slower release. On the other hand, a more loosely packed network with larger pores enables a more rapid release owing to increased availability and effectiveness of diffusion pathways, resulting in favorable transport phenomena, that is faster movement of drug molecules through the matrix and into the surrounding environment. These characteristics are common in hydrogel-based wound dressings designed for controlled drug release [53,56], underscoring the potential of BC as a versatile platform for long-term therapeutic applications.

In all cases, it is shown that the BC matrices offer the advantage of customizable release profiles depending on the specific wound care needs. Glycerol-derived BC may be optimal for scenarios requiring more rapid and consistent release, while arabinose and raffinose-based BC, with its slower release rate, provides prolonged pain management, particularly suited for chronic conditions. The ability to fine-tune BC’s drug release behavior through the choice of carbon source highlights its utility in creating wound dressings that cater to various clinical requirements, ensuring optimal therapeutic outcomes over extended periods. The three tested BC, with their sustained drug release, demonstrate potential for improving wound care, reducing the need for frequent reapplication, and enhancing patient compliance.

In conclusion, the drug release of glycerol, arabinose, and raffinose-based BCs underscores their suitability for long-term wound management. Despite differences in their release profiles, both offer controlled, extended release over 14 days, providing tailored options for either more rapid or more gradual therapeutic interventions.

#### 3.2.2. Swelling Capacity and Reusability of FD BC in Wound Care

**Figure 7:**
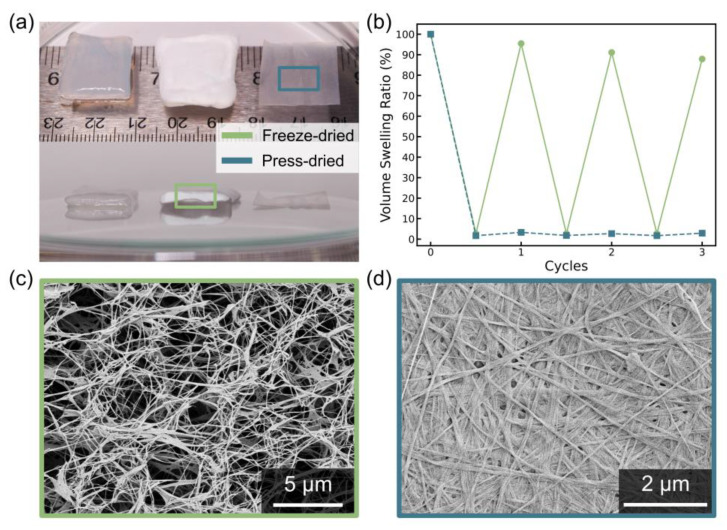
Photo showcasing the profiles of hydrated glycerol-derived bacterial cellulose (BC) from 9-day culture, freeze-dried BC and press-dried BC (**a**), cyclic swelling–drying curves of freeze-dried and press-dried BC (**b**), representative SEM image of the cross-section of freeze-dried BC (**c**) and top surface of press-dried BC (**d**).

The volume swelling ratio results highlight the superior recyclability and fluid retention capacity of FD BC samples compared to their PD BC counterparts, particularly for the GLY 9 sample, which was selected for its favorable lidocaine release profile over 14 days. As demonstrated in Figure 7a,b, FD BC maintained around 90% of its swelling capacity throughout multiple cycles, while PD BC exhibited a sharp decline after the first cycle. This behavior can be attributed to the preservation of the fiber network during freeze-drying, where the porous, three-dimensional architecture of the BC is maintained, preventing the collapse of the fibers, as demonstrated in Figure 7c,d [25]. This porous structure enables fluid uptake and retention, essential properties for applications in wound dressings that need to manage dynamic exudate levels during healing [51]. In contrast, press-drying leads to irreversible aggregation of cellulose fibers due to the formation of strong intermolecular hydrogen bonds [25], producing a denser and less porous structure that struggles to retain water after dehydration [57]. Although multiple SEM micrographs were taken for the pellicles from each modified HS medium, we only show a representative of each drying method here, with additional images provided in Figures S3 and S4. Previous studies further emphasize these findings that FD BC exhibited significantly better rehydration capacity, swelling ratio and structural stability compared to other drying processes [25,57]. The freeze-drying process effectively preserves the pore structure, enabling BC to repeatedly swell and deswell without significant loss in performance.

The FD BC, as demonstrated with GLY 9, shows superior rehydration performance, which is critical for applications in wound dressings. In a wound environment, the ability to undergo multiple swelling–deswelling cycles allows the dressing to handle varying exudate volumes effectively throughout the healing process [52]. Initially, a wound may produce abundant exudates that require rapid absorption, while later stages of healing demand moisture retention to prevent desiccation. The porous structure preserved in the FD BC enables it to absorb fluids efficiently, while maintaining a moist wound environment conducive to epithelial migration and tissue regeneration [20]. This repeated capacity to reabsorb fluid without significant loss of performance underscores the advantage of using FD BC in dynamic wound care situations, as it can adapt to the changing needs of the healing process, which is critical for epithelial cell migration and wound closure [20].

The superior performance of FD BC highlights its potential as a more effective material for hydroactive wound dressings, capable of handling the dynamic nature of wound exudates and promoting faster, more efficient healing by maintaining an optimal moist environment [52].

## 4. Conclusions

This study highlights the critical influence of carbon source selection in shaping the mesostructure, yield, and drug release performance of BC foams. The comparison between glycerol, raffinose, arabinose, glucose, and sucrose revealed that the molecular characteristics of each carbon source distinctly influence the BC fibrous network and crystallinity. Glycerol-based BC exhibited the highest yield, reaching 20.35 g of wet BC by day 14, while sucrose-based BC had the lowest yield at 8.97 g, underscoring the impact of carbon source on BC production. Crystallinity index values were highest for glycerol-based BC (exceeding 80%), with raffinose, arabinose, and sucrose showing increasing trends, reaching 79.4%, 79.8%, and 77.0%, respectively, by day 12. The cumulative lidocaine release from glycerol-based freeze-dried BC foam reached over 45% by day 14, compared to 37% and 27% for arabinose and raffinose-based BC, respectively, demonstrating the role of BC fiber geometry and network morphology in drug release profiles. Glycerol-based BC showed a network with larger pores and thicker fibers, which facilitated higher permeability and consequently the most rapid and sustained lidocaine release, positioning it as an optimal candidate for long-term wound care applications. In contrast, raffinose-based BC, due to its smaller average pore size, exhibited slower drug diffusion, making it more suitable for conditions requiring controlled, extended drug release, such as chronic wound management.

These findings highlight the tunability of BC properties through carbon source selection, allowing the development of BC-based materials tailored to specific biomedical applications. By fine-tuning the carbon source, BC can be customized for targeted wound care solutions, ranging from fast-acting drug release for acute wounds to sustained, controlled delivery for long-term therapeutic interventions. Future research will focus on scaling production and expanding the application of BC foams in drug delivery and tissue engineering, further enhancing their potential as versatile platforms for advanced biomedical applications.

## Data Availability

The original contributions presented in the study are included in the article/Supplementary Material, further inquiries can be directed to the corresponding author.

## Supplementary Materials

The following supporting information can be downloaded at: https://www.mdpi.com/article/10.3390/polym16213021/s1, Figure S1: One-dimensional radial WAXS profile (azimuthal average) at 9 D growth time (a), 10 D growth time (b), 11 D growth time (c), and 12 D growth time (d).; Table S1: Calculated values of coherent scattering regions (CSR) dimensions of bacterial cellulose (BC) produced using different sugars determined by wide-angle X-ray scattering (WAXS) analysis, unit of measurement is angstrom (Å); Table S2. Attribution of FTIR peaks for the analyzed samples. FTIR spectra to identify cellulose functional groups; Figure S3. SEM images of the surface of bacterial cellulose (BC) samples at 9-day growth time (a), 10-day growth time (b), 11-day growth time (c), and 12-day growth time (d); Figure S4. SEM images of the cross-sections of freeze-dried bacterial cellulose (BC) samples. References [58,59,60,61,62,63,64,65,66,67,68,69,70,71] are cited in the Supplementary Materials.
