# Supplementary material for: Impact of Carbon Source on Bacterial Cellulose Network Architecture and Prolonged Lidocaine Release
Source: Polymers (Basel). 2024 Oct 28;16(21):3021. doi: 10.3390/polym16213021 (PMC11548197; doi:10.3390/polym16213021)
Supplement: Supplementary file 1 [file polymers-16-03021-s001.zip › polymers-3263521-supplementary.pdf]

polymers MDPI

Supplementary Material

Impact of Carbon Source on Bacterial Cellulose Network Architecture and Prolonged Lidocaine Release

Julia Amorim [1,2], Kuotian Liao [2], Aban Mandal [2], Andréa Fernanda de Santana Costa [3,4], Eleftheria Roumeli [2] and Leonie Asfora Sarubbo [3,5] *

[1] Rede Nordeste de Biotecnologia (RENORBIO), Universidade Federal Rural de Pernambuco (UFRPE), Rua Dom Manuel de Medeiros, s/n - Dois Irmãos, 52171-900, Recife, Pernambuco, Brasil; julia.didier@ufrpe.br
[2] Department of Materials and Science and Engineering, University of Washington (UW), 2110 Mason Road, Roberts Hall 302, Seattle, WA, 98195; timliao@uw.edu; abanm@uw.edu; eroumeli@uw.edu
[3] Instituto Avançado de Tecnologia e Inovação (IATI), Rua Potyra, n. 31, Prado, 50751-310, Recife, Pernambuco, Brasil
[4] Centro de Design Comunicação, Centro Acadêmico da Região Agreste, Universidade Federal de Pernambuco (UFPE), Av. Marielle Franco, s/n - Nova Caruaru, Caruaru, Pernambuco 50670-90, Brasil; andrea.santana@ufpe.br
[5] Escola de Tecnologia e Comunicação, Universidade Católica de Pernambuco (UNICAP), Rua do Príncipe, n. 526, Boa Vista, 50050-900, Recife, Pernambuco, Brasil
* Correspondence: leonie.sarubbo@unicap.br; Tel.: +55-81- 21194000

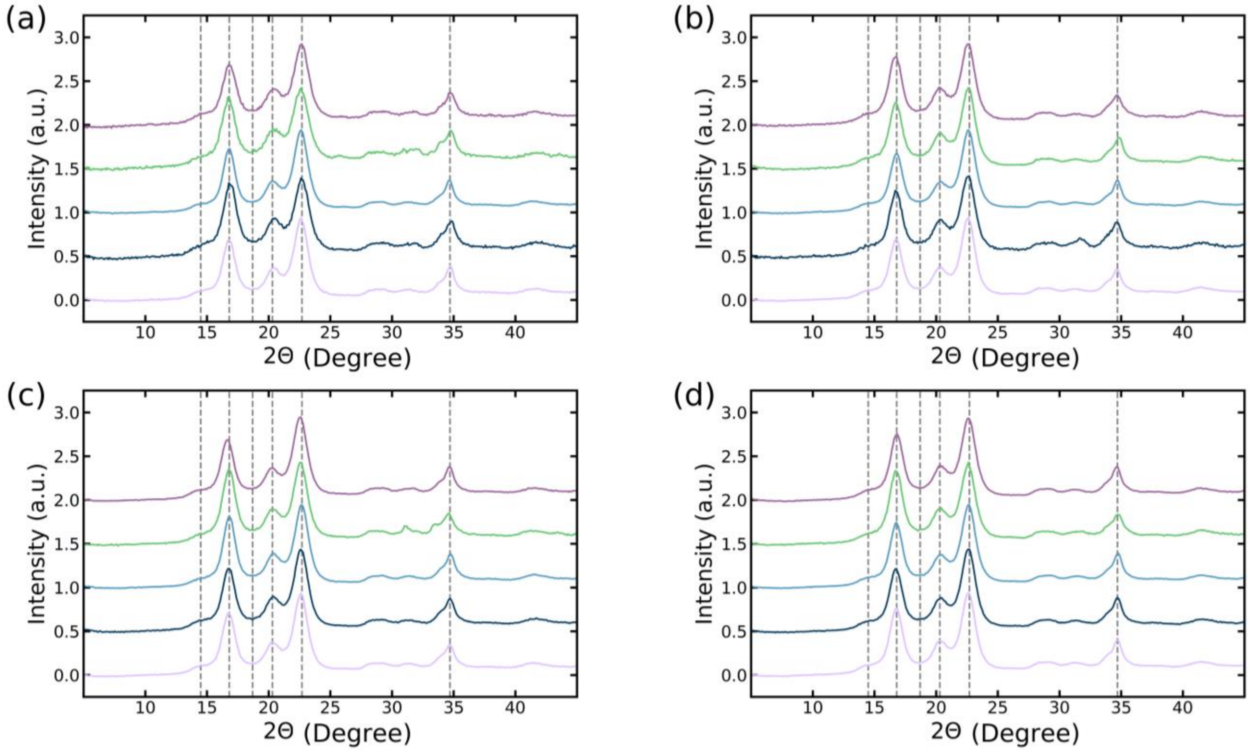

Figure S1. One-dimensional radial WAXS profile (azimuthal average) at 9D growth time (a), 10D growth time (b), 11D growth time (c), and 12D growth time (d).

Table S1. Calculated values of coherent scattering regions (CSR) dimensions of bacterial cellulose (BC) produced using different sugars determined by wide-angle X-ray scattering (WAXS) analysis[1], unit of measurement is angstrom (Å)

Sample	D_{100}	D_{010}	$D_{01\bar{2}}$	D_{110}	$D_{01\bar{4}}$
GLY 9	51.49	65.88	54.11	61.08	86.98
GLY 10	49.99	65.60	51.21	61.76	83.63
GLY 11	52.43	65.05	51.41	61.51	84.58
GLY 12	48.09	64.83	51.84	60.04	86.77
ARA 9	47.70	60.55	47.86	56.50	75.15
ARA 10	50.38	62.71	47.80	58.95	85.02
ARA 11	46.62	64.47	50.49	60.96	83.71
ARA 12	49.01	64.90	51.49	60.88	87.01
GLU 9	51.77	69.93	52.86	61.28	86.99
GLU 10	49.94	67.97	50.66	59.88	87.00
GLU 11	49.53	69.49	53.19	62.31	87.01
GLU 12	47.02	66.86	49.77	59.99	87.01
SUC 9	44.67	60.40	43.06	55.84	82.72
SUC 10	42.08	64.15	47.14	62.43	84.94
SUC 11	49.47	64.19	49.27	61.26	77.40
SUC 12	47.54	62.72	47.88	59.38	79.56
RAF 9	47.03	58.63	44.09	57.42	86.34
RAF 10	46.22	62.60	46.62	60.56	75.92
RAF 11	48.95	65.02	51.34	59.89	87.01
RAF 12	47.66	65.92	51.15	60.86	87.00

[1] Samples are labeled according to the carbon source used (e.g., GLY for glycerol, ARA for arabinose) and the day of harvest (e.g., GLY 9 refers to BC synthesized with glycerol and harvested on day 9).

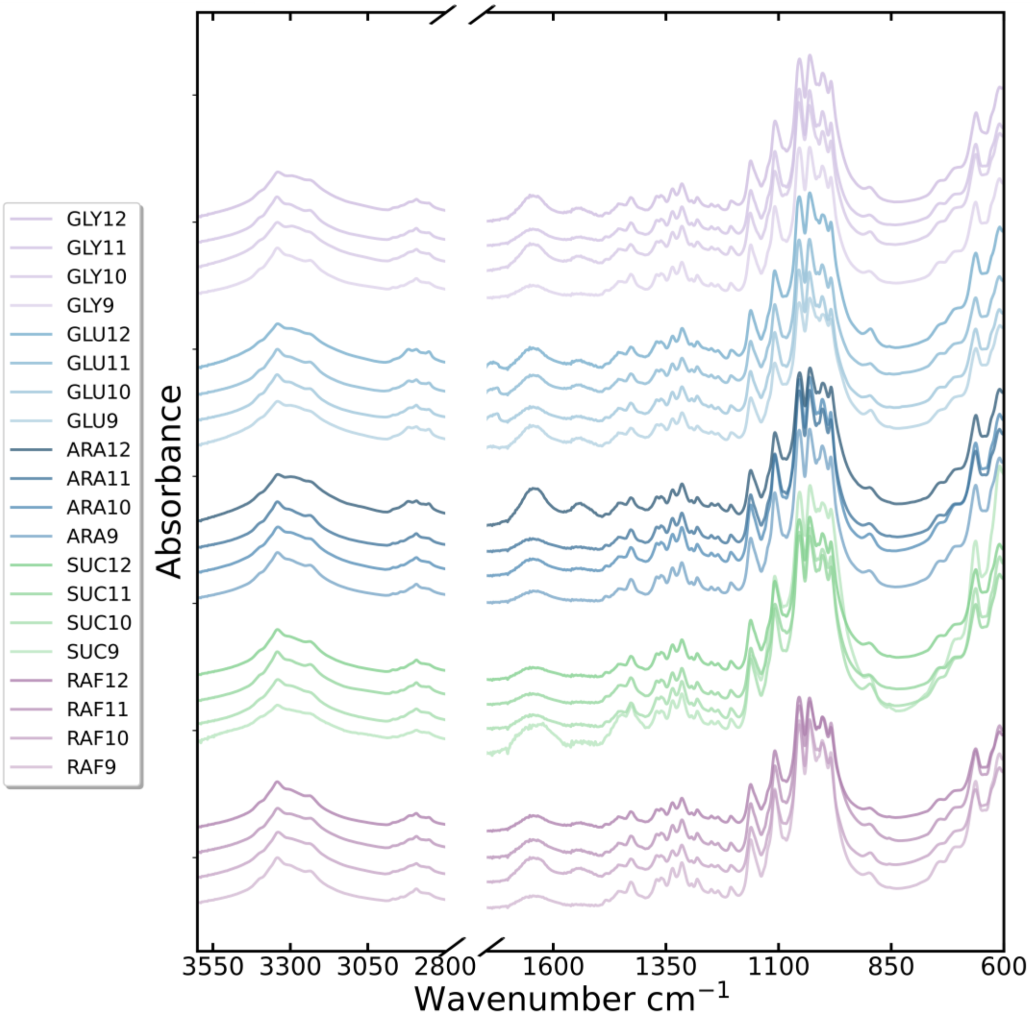

Figure S2. FTIR spectra highlighting key functional groups in our cellulosic samples.

Table S2. Attribution of FTIR peaks for the analyzed samples.

Peak Location (cm^{-1})	Vibration	Attribution	References
3400	(O-H)	6O-H interchain h-bond to 3O	[1]
3340	v(O-H)	2,3,6O-H coupled vibration	[1]
3280	v(O-H)	2O-H cellulose Iβ intrachain H-bond to 6O	[1]
3230	v(O-H)	2O-H cellulose Iα intrachain H-bond to 6O	[1]
2920	v_{as}(C-H)	Methyl and methylene groups	[2]
2892	v(C-H)	CH_2 and CH_3 groups	[3]
2850	v_{as}(C-H)	CH_2	[4]
1733	v(C=O)	Lipids from cell remnants	[5]
1651	v(C=O) or δ(O-H)	Amide I from protein in cell residue or rocking vibration in residual water	[6,7]
1549	v(C-N) or ϱ(N-H)	Amide II from protein in cell residue	[6]
1451	ϱ(O-H)	In plane bending of hydroxyl groups	[8]
1427	δ(C-H)	In plane scissoring in CH_2	[8–11]
1365	w(C-H) and/or δ_{as}(C-H)	Out of plane rocking/wagging in CH_2	[12]

1335	ϱ(O-H) or δ(C-H)	C-H bending or in plane bending of hydroxyl	[3]
1314	w(C-H)	Out of plane wagging in CH_2	[12,13]
1205	v(C-O-C)	Symmetric stretching of C-O-C	[13]
1161	v_{as}(C-O-C)	Asymmetric stretching at the glycosidic link	[12]
1108	v(C-C)	C-C in the ring symmetric stretching	[14]
1054	δ(C-O)	Bending of C-O bonds to hydroxyl groups	[3,13]
1031	v(C-O)	Mainly of C6-O6 hydroxyl group	[3,12]

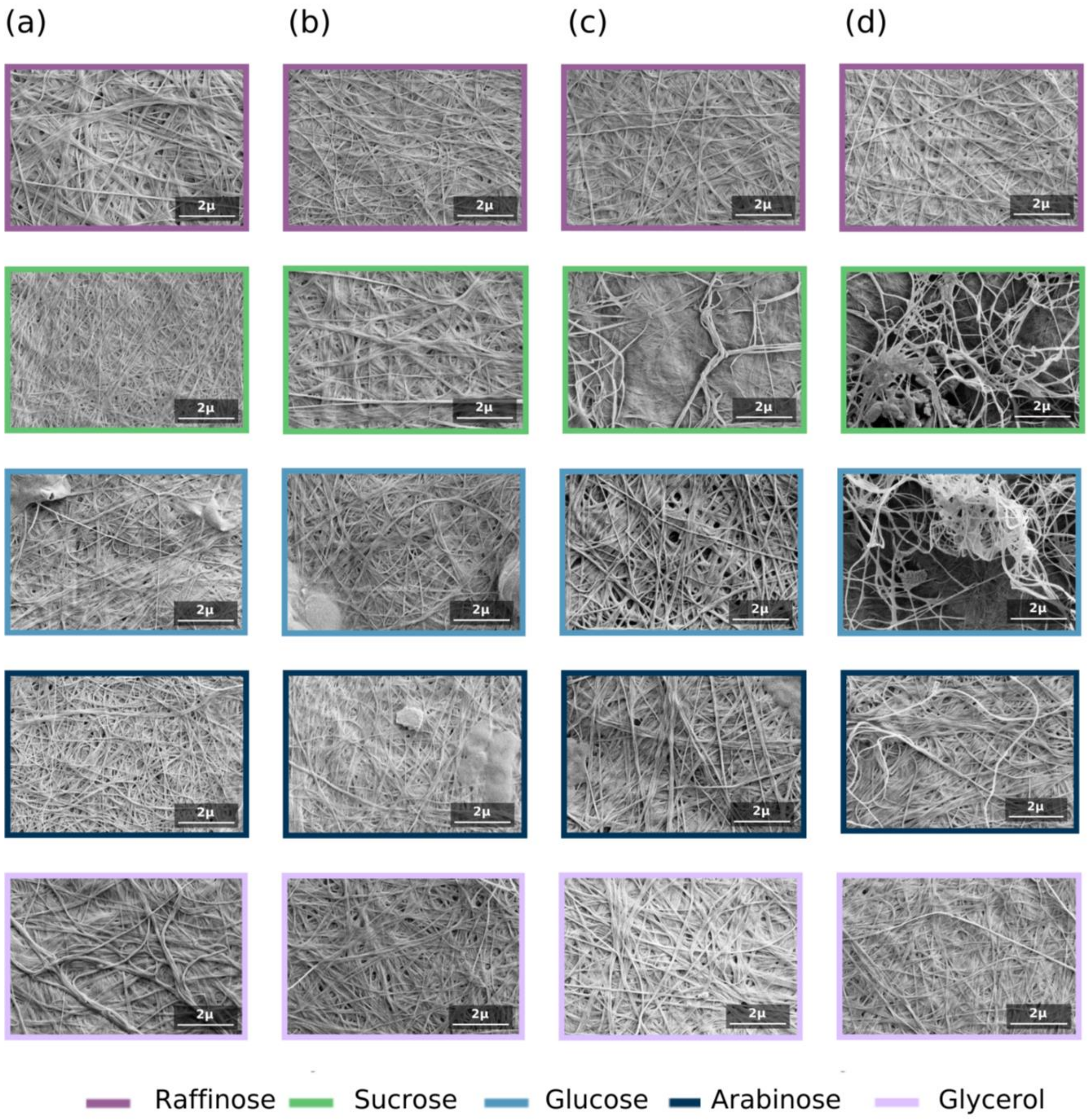

Figure S3. SEM images of the surface of bacterial cellulose (BC) samples at 9-day growth time (a), 10-day growth time (b), 11-day growth time (c), and 12-day growth time (d).

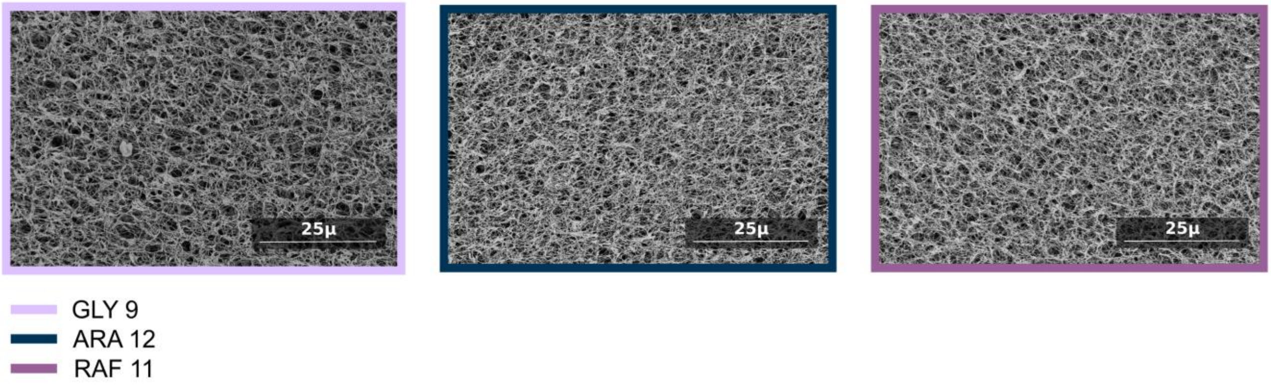

Figure S4. SEM images of the cross-sections of freeze-dried bacterial cellulose (BC) samples.

References

1. Lee, C.M.; Kubicki, J.D.; Fan, B.; Zhong, L.; Jarvis, M.C.; Kim, S.H. Hydrogen-bonding network and OH stretch vibration of cellulose: Comparison of computational modeling with polarized IR and SFG spectra. *J. Phys. Chem*. B 2015, 119 (49), 1021–1028. doi:10.1021/acs.jpcb.5b08015.
2. Moosavinejad, S.M.; Madhoushi, M.; Vakili, M.; Rasouli, D. Evaluation of degradation in chemical compounds of wood in historical buildings using FT-IR and FT-Raman vibrational spectroscopy. *Maderas Cienc. Tecnol.* 2019, 21 (3), doi:10.4067/S0718-221X2019005000310.
3. Wang, S.-S.; Han, Y.-H.; Ye, Y.-X.; Shi, X.-X.; Xiang, P.; Chen, D.-L.; Li, M. Physicochemical characterization of high-quality bacterial cellulose produced by Komagataeibacter sp. strain W1 and identification of the associated genes in bacterial cellulose production. *RSC Adv.* 2017, 7 (45145-45155), doi:10.1039/C7RA08391B.
4. Gea, S.; Reynolds, C.T.; Roohpour, N.; Wirjosentono, B.; Soykeabkaew, N.; Bilotti, E.; Peijs, T. Investigation into the structural, morphological, mechanical and thermal behaviour of bacterial cellulose after a two-step purification process. *Bioresour. Technol.* 2011, 102 (19), 9105–9110, doi:10.1016/j.biortech.2011.07.001.
5. Fuller, M.E.; Andaya, C.; McClay, K. Evaluation of ATR-FTIR for analysis of bacterial cellulose impurities. *J. Microbiol. Methods* 2018, 144, 145–151, doi:10.1016/j.mimet.2017.10.017.
6. Mallamace, F.; Corsaro, C.; Mallamace, D.; Vasi, S.; Vasi, C.; Dugo, G. The role of water in protein's behavior: The two dynamical crossovers studied by NMR and FTIR techniques. *Comput. Struct. Biotechnol. J.* 2015, 13, 33–37, doi:10.1016/j.csbj.2014.11.007.
7. Kačuráková, M.; Belton, P.S.; Wilson, R.H.; Hirsch, J.; Ebringerová, A. Hydration properties of xylan-type structures: An FTIR study of xylooligosaccharides. *J. Sci. Food Agric*. 1999, 77(1), 38–44, doi:10.1002/(SICI)1097-0010(199805)77:1<38::AID-JSFA999>3.0.CO;2-5.
8. Gierlinger, J.; Gollier, C. *Socially Efficient Discounting under Ambiguity Aversion*; Institute d'Economie Industrielle: Toulouse, France, 2008.
9. Barud, H.S.; Souza, J.L.; Santos, D.B.; Crespi, M.S.; Ribeiro, C.A.; Messaddeq, Y.; Ribeiro, S.J.L. Bacterial cellulose/poly(3-hydroxybutyrate) composite membranes. Carbohydrate *Polymers* 2011, 83(3), 1279–1284. doi:10.1016/j.carbpol.2010.09.049
10. Zhou, L.L.; Sun, D.P.; Hu, L.Y.; Li, Y.W.; Yang, J.Z. Effect of addition of sodium alginate on bacterial cellulose production by Acetobacter xylinum. *J. Ind. Microbiol. Biotechnol.* 2007, 34(7), 483. doi:1 0.1007/s10295-007-0218-4
11. Dammström, S., Salmén, L., & Gatenholm, P. The effect of moisture on the dynamical mechanical properties of bacterial cellulose/glucuronoxylan nanocomposites. *Polymer* 2005, 46(23), 10364–10371. doi:10.1016/j.polymer.2005.07.105
12. Kačuráková, M., Smith, A.C., Gidley, M.J., & Wilson, R.H. Molecular interactions in bacterial cellulose composites studied by 1D FT-IR and dynamic 2D FT-IR spectroscopy. *Carbohydr. Res.* 2002, 337(12), 1145–1153. doi:10.1016/S0008-6215(02)00102-7
13. Gierlinger, N.; Goswami, L.; Schmidt, M.; Burgert, I.; Coutand, C.; Rogge, T.; Schwanninger, M. In situ FT-IR microscopic study on enzymatic treatment of poplar wood cross-sections. *Biomacromolecules* 2008, *9* (8), 1021–1028. doi:10.1021/bm800300b.
14. Movasaghi, Z.; Rehman, S.; Rehman, I.U. Fourier transform infrared (FTIR) spectroscopy of biological tissues. *Appl. Spectrosc. Rev.* 2008, 43 (2), 134–179. doi:10.1080/05704920701829043.

Disclaimer/Publisher's Note: The statements, opinions and data contained in all publications are solely those of the individual author(s) and contributor(s) and not of MDPI and/or the editor(s). MDPI and/or the editor(s) disclaim responsibility for any injury to people or property resulting from any ideas, methods, instructions or products referred to in the content.
